# An Engineering Perspective of External Cardiac Loop Recorder: A Systematic Review

**DOI:** 10.1155/2016/6931347

**Published:** 2016-10-31

**Authors:** Avvaru Srinivasulu, N. Sriraam

**Affiliations:** ^1^Department of Electronics and Instrumentation Engineering, GITAM University, Bangalore Campus, Bangalore, India; ^2^Center for Medical Electronics and Computing, M.S. Ramaiah Institute of Technology, Bangalore, India

## Abstract

External cardiac loop recorder (ELR) is a kind of ECG monitoring system that records cardiac activities of a subject continuously for a long time. When the heart palpitations are not the frequent and nonspecific character, it is difficult to diagnose the disease. In such a case, ELR is used for long-term monitoring of heart signal of the patient. But the cost of ELR is very high. Therefore, it is not prominently available in developing countries like India. Since the design of ELR includes the ECG electrodes, instrumentation amplifier, analog to digital converter, and signal processing unit, a comparative review of each part of the ELR is presented in this paper in order to design a cost effective, low power, and compact kind of ELR. This review will also give different choices available for selecting and designing each part of the ELR system. Finally, the review will suggest the better choice for designing a cost effective external cardiac loop recorder that helps to make it available even for rural people in India.

## 1. Introduction

Norman J. Holter (1914–1983), the famous American biophysicist, introduced a remote cardiac telemetry first time in the 1940s [1]. The Holter system was developed for home ECG monitoring of patients with suspected cardiac arrhythmias. The original Holter monitor had analog patient interface electronics, a 75 lb backpack with a reel-to-reel FM tape recorder, and large batteries. It was the first monitoring system that could record single ECG lead 24–48 hours [2] and analyse ambulatory ECG data outside a standard hospital or outpatient care setting. At present, the Holter monitors are available in the market with the cost of about $369–$2490 [3] depending on their features and the cost of Holter test is around $175–$250 [4] if it is interpreted by a cardiologist. The clinical need to monitor ambulatory ECG has resulted in advances in technology that now allow us to monitor heart rhythms remotely through a wide variety of devices, including ambulatory external monitors and implantable event recorders.

Implantable/insertable loop recorder (ILR) was developed first time by Medtronic's Reveal [5] (the world's first implantable diagnostic device). The Reveal ILR detects ECGs during the actual episode, which may allow physicians to take decisions or confirm an abnormal heart rhythm more definitively. Because it could be worn continuously for 14 months, the likelihood of capturing heart rhythm during an infrequent episode was probable. The cost per diagnosis using ILR is around $6,158 [6]. The cause of seizure-like symptoms or related symptoms was diagnosed with the Reveal ILR that may also result in fewer physician and emergency room visits and reduce the number of tests involved when trying to diagnose their cause. Most importantly, diagnosing the cause helps in early treatment effectively. Even though ILR was useful in monitoring of ECG for the detection of abnormal episodes, it had some disadvantages that include the following: (1) a minor surgical procedure is needed, (2) there is always difficulty in differentiating supraventricular from ventricular arrhythmias, (3) under- or oversensing may exhaust the memory of the ILR, and (4) cost of the device is more.

To overcome the limitations of both the Holter and ILR, an intermittent patient- or event-activated recorder was developed. This is also referred to as event monitor or external loop recorder (ELR). The ELR is smaller than Holter in size and is attached to the patient through chest electrodes and records the data when it is activated by the patient or by an automatic trigger that detects irregular heart rates. It is used for monitoring up to 14–30 days. The cost of ELR is $627 and cost per diagnosis using ELR is around $265,9 [7]. The use of ELR avoids the surgical implantation of electrodes. But the activation of the device every time by the patient is difficult unless the autotrigger is used. The autotrigger activates the device as it is programmed which is built into the monitor. Therefore during infrequent symptoms, there is a more chance of missing the activation of the device. This may not give enough information for effective diagnosis. After recording using any of the above-mentioned systems, the data is sent to the central monitoring station where the data is loaded in the computer and analysed. Finally, the reports are sent to the doctor for a final decision or for further tests to detect and confirm the disease. As mentioned above, ELR is providing the noninvasive diagnosis by long-term monitoring. Even the cost of design is less, the ELR test cost is high. Further, the recorded data has to be sent to the specialist to analyse the data which increase the cost further. And there is no option for autosending the recorded data or analysed data to the doctor. The comparison among Holter, ILR, and ELR is given [8] in Table 1.

In Table 2 some of the available ILR and ELR products are given [5, 9–16].

The mentioned products in Table 2 are very expensive and most of them are not significantly available in India. Distributors are there all over India, but they are providing only a few products like Medtronic SpiderView, SEEQ MCT, Piix NUVANT MCT, GE Healthcare MARS, SEER 1000, SEER Light, Omron HCG801, and BPL cardiac loop recorder monitor. Therefore, a systematic review on internal parts of ECG monitoring system is required to design a cost effective ambulatory ECG monitoring system with an accurate measurement, portable and wearable one as explained in the following sections.

## 2. Designing of External Cardiac Loop Recorder

**Figure 1:**
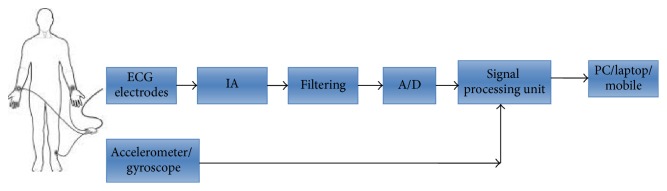
Block diagram of external cardiac loop recorder.

The design of external cardiac loop recorder consists of ECG electrodes, instrumentation amplifier, filtering, analog to digital converter, and signal processing unit. PC/laptop/mobile is also used to analyse the data. The major blocks and connection of them are shown in Figure 1.

The ECG signal is acquired from the chest electrodes and is amplified by the instrumentation amplifier. The amplified signal is filtered by the suitable filter to remove the noise. Mostly band pass filter is used for noise removal. Later, an analog to digital converter converts the filtered signal into a digital form which is suitable to process signal by the processor. A signal processing unit is used for processing and feature extraction of the signal to find the normal and abnormal conditions of the patient. For the effective detection of the abnormal conditions during daily activities accelerometer and/or gyroscope is also used along with the chest electrodes. By correlating the signals from chest electrodes and accelerometer/gyroscope, the abnormality of the patient can be defined. The signal processing unit is connected to the PC/laptop/mobile or system on chip (SoC) where the open source software is installed and used for displaying, processing, and saving the data. Further, communication with the doctor can be provided using wireless technology which helps to develop the smart city. The comparative study of each block is explained in following sections.

### 2.1. ECG Electrodes

**Figure 2:**
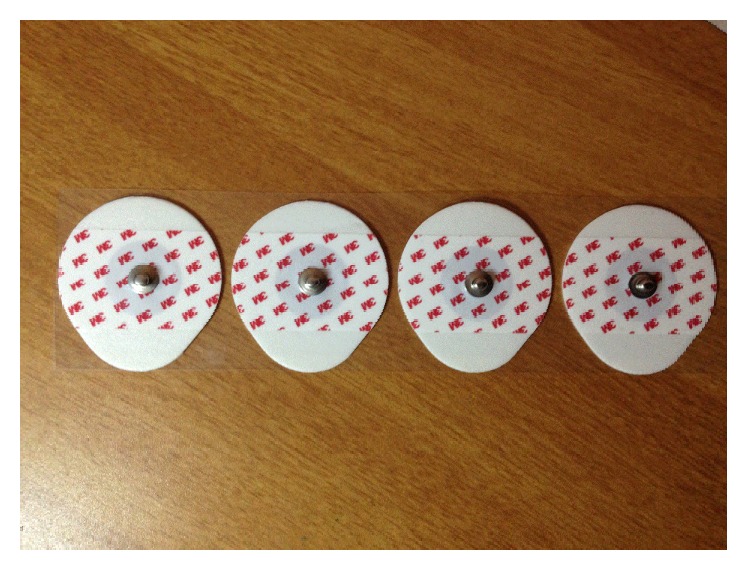
Disposal Ag/AgCl electrodes.

Basically, disposal electrodes that may be Ag/AgCl gel type wet sensors or dry sensors are used for acquiring biopotentials from heart. The gel type disposable electrodes have a circular contact. The close electrode placement is allowed by small vinyl backing where necessary and a slightly less firm adhesive allows ouchless removal. The electrodes incorporate liquid electrolyte gel and moderately high chloride salt concentration for quick and accurate readings. These disposable electrodes shown in Figure 2 provide the same signal transmission as reusable electrodes, with added convenience. Each peel and stick electrode is pregelled and designed for one use only. It is very cost effective compared to other electrodes. It is easily attachable to the subject himself/herself and there will not be any assistance needed. The placement of electrodes is also simple and only three electrodes at a time are required for two lead ECG acquisition systems as one electrode is the reference. These electrodes can be used for longer periods depending on the comfort level of the subject.

**Figure 3:**
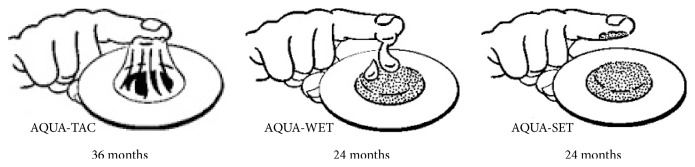
SKINTACT electrodes.

SKINTACT electrodes [73] shown in Figure 3 are available in the market with three different gels: AQUA-TAC electrode with solid adhesive gel provides 100% contact with skin surface, AQUA-WET electrode with liquid gel provides fast pickup of ECG signal which is preferred for short term monitoring, and AQUA-SET electrode with solid wet gel is used for long-term monitoring.

**Figure 4:**
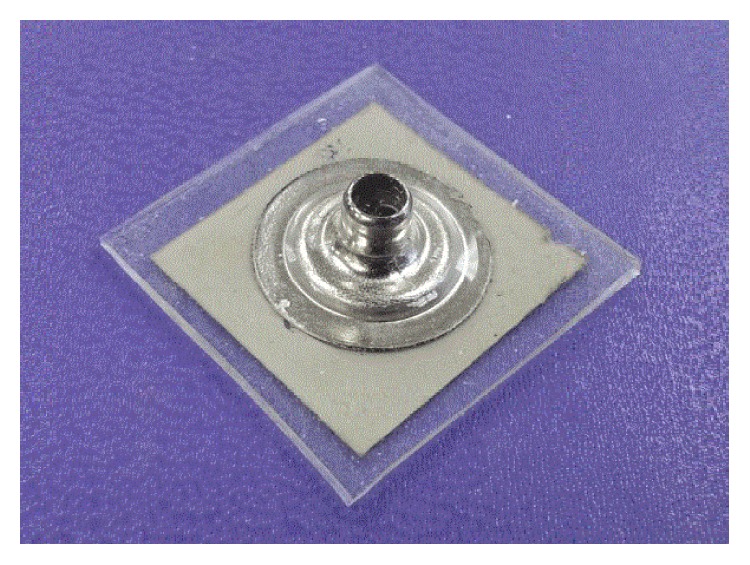
Dry electrode.

North Carolina State University researchers [74] had developed a new dry sensor shown in Figure 4 for long-term ECG and EMG monitoring. This device has relied on elastic conductors made from silver nanowires embedded in a pliable polymer.

**Figure 5:**
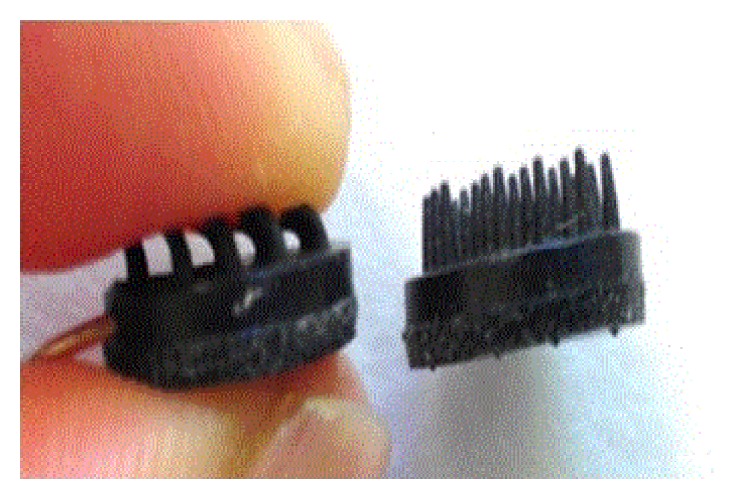
Polymer dry electrodes.

Imec and Holst Centre introduced the polymer dry electrodes [75] shown in Figure 5 fabricated from ethylene propylene diene monomer (EPDM) rubber which offers a high user comfort and high conductivity.

**Figure 6:**
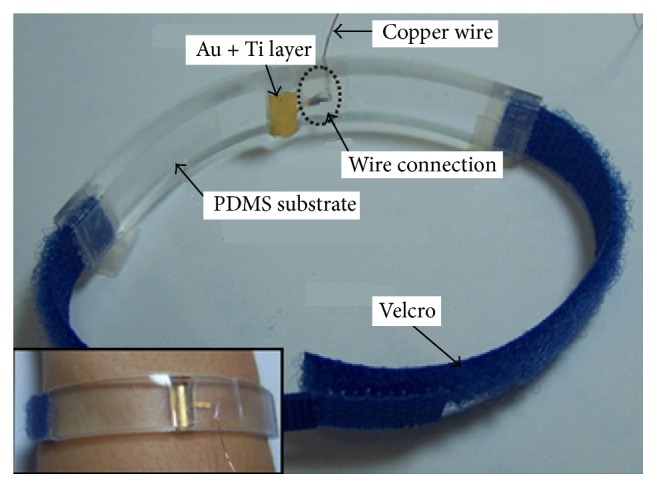
PDMS surface electrode.

PDMS (polydimethylsiloxane) based surface electrode shown in Figure 6 was designed [76] for the long-term and unsupervised monitoring. This electrode did not show negative influence on skin even it was worn for one week.

**Figure 7:**
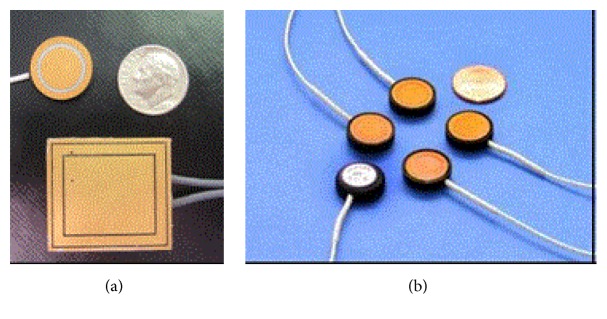
(a) QUASAR IBEv1 electrodes; (b) QUASAR IBEv2 electrodes.

Apart from wet and dry electrodes, there are noncontact electrodes called capacitive electrodes. These were fabricated on silicon with a thermally grown silicon dioxide as the dielectric layer. Dry capacitive electrodes were used for short-term ECG monitoring [77]. A new class of bioelectric sensors was developed by quantum applied science and research (QUASAR) in 2002. These electrodes were capacitively coupled with the body by incorporating the sensors into shirts, elastic belts, and glasses. The QUASAR two-generation electrodes are shown in Figure 7(a). The first-generation electrode IBEv1 is a larger, square sensor (1′′  ×  1′′) used to measure bioelectric potentials through T-shirt [78]. The second-generation electrode IBEv2 was developed as a small circular shape sensor shown in Figure 7(b).

### 2.2. Accelerometers and Gyroscopes

Accelerometers and gyroscopes are also used along with dry or wet sensors for cancelling muscle contraction interferences, to measure heart rate under different activities like stress, movements, and so forth. The accelerometer is a 3-axis one. It is used in tilt-sensing applications, as well as dynamic acceleration resulting from motion or shock to measure the static acceleration of gravity. In previous work done the people used the accelerometer for different purposes. In previous work done, the people used the accelerometer for different purposes. ADXL335 triaxial accelerometer [9, 79] and triaxial accelerometer MotionPodTM by MOVEA were used for removal of motion artefacts. SDI1221, a low cost, integrated 1-axis accelerometer, was used in zero to medium frequency instrumentation applications to provide extremely low noise (5 *μ*g/√Hz) [80]. A triple axis accelerometer [81–83] and MMA7260QT [84] were used in telehealth monitoring. ADXL330 was used in deciding of the cardiac disease [85, 86]. Bosch BMA180 accelerometer was used in human behaviour tracing [87]. A triple axis accelerometer [81, 83, 88–90], ADXL345 [91], and ADXL330 [92] were used in activity recognition. MC301 made by Wacoh was used in ambulatory monitoring to find human posture and walking velocity [93]. MMA8451Q (Austin, TX, USA), a triple axis, low power, capacitive digital accelerometer (freescale semiconductor) [94], a triaxial accelerometer (patch sensor device designed by Vital Connect, Inc. (Campbell, CA)) [95], and inbuilt on-board 3-axis accelerometer SCA3000 [96] were used in extraction of respiratory rate. And also a triaxial accelerometer was used to measure the body movements [90] or daily stress [97] and for left ventricular functions monitoring [98]. A triaxial gait accelerometer MMA7260Q (freescale semiconductor, Austin, TX, USA) [99], piezoelectric foils [100], and Pegasus activity monitors developed by ETB, UK, were used for time-frequency analysis of heart rate. Triaxial accelerometer ADXL335 [9, 79] and MotionPodTM by MOVEA [98] were used as the reference for removing motion artefact by adaptive filtering algorithm (LMS or ANC) in acquiring of ECG during treadmill exercise. Apart from these, a triaxial accelerometer (LlS344ALH, ST Microelectronics) was used for seismocardiography.

**Figure 8:**
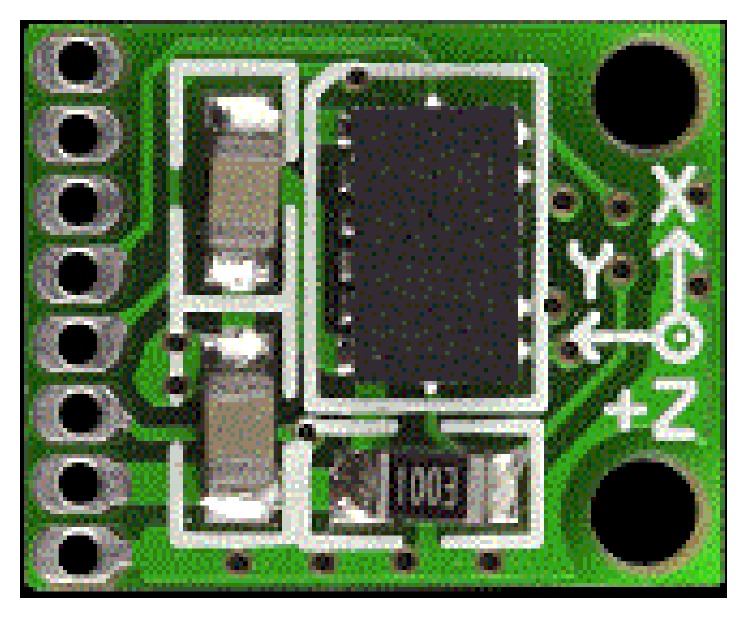
3-axis accelerometer ADXL345.

Among all the accelerometers mentioned in Table 3, the model ADXL345 shown in Figure 8 can be selected because of less power consumption and better full scale range with 2–3.6 V supply voltage. In ECG monitoring, the accelerometer is used to get the change in acceleration due to body movements during daily activities. This is helpful in detecting the arrhythmias. Finally, the heart rate measured by disposal electrodes and the accelerometer readings will be correlated. Using this information alerts or notifications are sent.

The gyroscope is used to find the tilt in position when there is motion in the body. This is required for monitoring of ECG during daily activities. In previous work, gyroscopes were used in different applications like L3G4200D gyroscope used for head movement tracking along with accelerometer and magnetometer [17]. Gyroscope and accelerometer inbuilt MEMS chip [101] were used in robotic arm control by detecting the motion of arm [102] and vehicle speed control [103]. Ring laser gyroscope [104] and microgyroscope [105] are advanced gyros used for various applications. In cardiac applications, gyroscope was used for monitoring electric and mechanical functioning of heart [106] (gyro developed by Zimpher Technology and Shimmer Research was used in [107]) means, heart rate [108], rotational velocity of foot [108], emotional eating (2-axis gyro was used), human posture and walking velocity (ENC03J developed by Murata Manufacturing Co. Ltd., Kyoto, Japan, was used in [18]), stride strength and walking velocity (ENV05S developed by Murata Manufacturing Co. Ltd., Kyoto, Japan, was used), muscle contractions (vibrating disc piezoelectric gyroscope was used in [19]), and motion processing in handsets (InvenSense MPU-3000 3-axis MEMS gyroscope was used).

The differences between gyroscope and accelerometer are given in Table 5 that help in the selection of gyroscope or accelerometer or both for ECG monitoring systems.

In order to differentiate the ECG signal due to heart activity from the patient's daily life activities, accelerometer and gyroscope alone are not sufficient. Therefore, it is suggested to use both accelerometer and gyroscope to find daily activities of patient effectively.

### 2.3. Placement of Electrodes

The placement of electrodes on the body varies based on type of wearable design. For different wearable types, placement of electrodes according to the previously proposed designs is given in Table 6.

### 2.4. Instrumentation Amplifier (IA)

#### 2.4.1. Mostly Used IA ICs

There are a number of instrumentation amplifier ICs available in the market suitable for ECG signal amplification. The use of IA in IC form is very easy and more convenient in ECG signal acquisition because of its small size and high noise immunity. The most widely used IA ICs were developed by Texas Instruments and Analog Devices. Texas Instruments ICs INA116 [109, 110], INA121 [111], and INA128 [112] were most widely used in ECG signal acquisition systems. INA116 provided high input impedance (1015 Ω) and the bandwidth of 0.38–44 Hz (±5%) with a single supply of 2 V; it was used for long time ECG monitoring of athletes [109]. It was also used in the designing of low noise EEG/ECG sensor circuit [110]. INA121 with a two-input voltage buffer as driving Right Leg (RL) circuit provided differential gain = 1000 from 0.05 Hz–100 Hz and common-mode gain = 0.06 at power-line frequency (50 Hz) that results in CMRR = 86 dB [111]. Analog devices ICs AD620 [113] and AD623 [114] were used for ECG signal acquisition and monitoring.

#### 2.4.2. Circuit Designs of IA

Basically the instrumentation amplifier is designed using operational amplifier which acts as voltage amplifier [115] that provided gain = 54.83 dB, CMRR = 141.61 dB, and bandwidth = 223 Hz [55]. A simple unity-gain buffer stage and differential amplifier stage with high input impedance [116] were used to design IA to have optimised low-frequency response, low power, and CMRR. The minimum input resistance of the amplifier required was obtained as 1.3 MΩ [117]. A composite stabilised amplifier with active current feedback at its input stage was used to reduce amplifier saturation problems and baseline drift [118] in off-the-shelf ECG amplifier for a continuous long duration. But the amplitude is not matched with that of standard (3 electrodes) voltage ECG amplifier. If RE < 50 kΩ, the bandwidth of the circuit will decrease below the bandwidth of the acceptable limit. DDA (differential difference amplifier) was used to lower the power consumption and keep the open loop gain to enough value. The AC coupled technique was used to reduce offset noise. DDA with AC coupled technique [61] provided power supply rejection ratio = 62 dB & CMRR = 150 dB at 10 Hz and with the preferred input noise at 5 *μ*V/Hz power consumption = 3.99 *μ*W at 1 Hz. To remove offset voltage and reduce 1/*f* noise, the low-frequency signal was to be eliminated. This was done by differential AC coupling network and the HP difference amplifier [119]. A design for remote electrocardiogram system, which consists of five stages ECG input, isolated amplifier, main amplifier, active BRF, and high order LPF with bandwidth 1 Hz–200 Hz [120], was used for ECG signal amplification and power supply (60 Hz) noise reduction.

Two-stage IA using operational transconductance amplifier (OTA) and common-mode feedback amplifier topology was used for common-mode amplifier noise reduction. This provided power consumption = 1.47 *μ*W and CMRR = 82 dB [56]. An IA with series combination of two OTAs (one is preamplifier and second is variable-gain amplifier) provided power consumption = 233 nW, bandwidth = 21 Hz, gain = 44.2 dB, and CMRR = 80 dB [60]. Flicker noise was removed by both chopper stabilised front end amplifier [121] and chopped capacitively coupled IA (CCIA) [122, 123]. Chopper technique which was implemented using folded cascode structure provided 36.44 dB of SNR in [54].

The instrumentation amplifier using the opamp for ECG signal acquisition cannot reduce noise much effectively. Therefore in order to solve this problem ECG amplifiers were designed using CMOS technology [56–63, 124–127] which also provide less power consumption and small area. The noise reduction in terms of CMRR obtained in different papers is mentioned in Table 7.

From Table 7, one can observe that most of the work reported was based on usage of same processing technology with different battery voltage. The work done in [62] was given better common-mode rejection ratio with a Monolithic CMOS current-mode instrumentation amplifier.

### 2.5. Filter

Filtering was required to remove the noise in ECG signal acquisition from electrodes followed by IA. The noise interferences were involved in many ways in ECG acquisition as its amplitude is less (in the order of mV) and variability of ECG segments durations. Muscle contractions, electrode movements during acquisition, base line wandering, and 60 Hz power supply noise were some of the significant noise interferences. And also, filtering was required to separate the segment of interest from the acquired ECG signal like P wave, R-peak, QRS complex, T wave, and ST segment. Here removal of noise interference was not considered in this paper. Different filters and their frequency range for different parameters acquisition used by previously proposed authors are given in Table 8 for selecting and deigning of required filter.

From Table 8, it is shown that the most of the authors used LPF and HPF or BPF for measuring almost any parameter. But the frequency range is not the same for all. It is different for different parameters. Therefore, the designer has to select the frequency range based on his/her segment of interest.

### 2.6. ADC

The ADC ICs such as 16-bit, 100-kSPS SAR ADC ADS83212 [33], 10-bit SAR ADC [30, 38, 128], and 24-bit ADS1292 [129] were used for analog to digital conversion of signal. But nowadays the signal processing development boards like Texas products ADS1298, ADS1191, ADS1192, ADS1194, ADS1196, ADS1198, ADS1291, ADS1299, ADS1298R, ADS1296R, ADS1296, ADS1294R, ADS1294, ADS1293, and ADS1291 that provide analog voltage 2.7 V–5.25 V and digital voltage 1.65 V to 3.6 V [130] and analog devices ADAS1000 (low power, 5-electrode ECG analog front end) and AD8232 (single-lead heart rate monitor analog front end) [131] are available with ADC inbuilt at significantly reduced size, power, and overall cost. Therefore, there is no need for external ADC to place.

### 2.7. Signal Processing Unit

Generally microcontroller board is used as signal processing unit to process the digital signal. This unit is further connected to PC/laptop to display the signals and measurements. It can also be used to communicate with other systems using transmitter and receiver. In previous designs proposed, for short term monitoring of ECG for 10 sec or 1-2 minutes MSP430 microcontroller was used [34, 71] and for long-term monitoring TI CC2530 system [37], CC2431 [132], DSP [128], DSP chip TMS320VC5509A [133], TMS320F2812 [134], TMDX5505eZDsp/VC5505eZdsp [33], MSP430 (monitoring for 45 days) [68], MSP430F5515 [129], MSP430F1232 [43], MSP430FG439 [135], MSP430F2418 [136], MSP430F5529 [66] (monitoring for 88 h) [39], MSP430F5419A (monitoring for 48 h) [137], ATmega8 [41], ATmega328 [42], Arduino UNO (ATmega328) [47], ATmega8L [28, 31, 32], Concerto MCU [65], Revitive Device [27], PIC18LF4620 [69], Altera EP2C35 Nios II soft-core CPU based FPGA [138], ARM9 [139], ADuC842 [140], C8051F021 [141], 32-bit ARM Cortex M0 CPU (monitoring for 24 h) [30], and STM32 chip as the system controller with ARM Cortex-M3 core (monitoring for 44 h) [67] were used.

### 2.8. Communication to PC/Laptop/Mobile Phone

#### 2.8.1. Need of Communication to PC/Laptop/Mobile Phone/Soc Network

After acquiring ECG signal, to display process and report the results of analysis to physician or doctor for diagnosis of the disease, there is a need for connecting to PC or Laptop. Mobile also can be used with specially designed apps.

#### 2.8.2. Available Communication Techniques

1. USB-SPI is generally used to connect the MC development board to PC. To display the signals and measurements MATLAB Simulink GUI or specially designed GUI is used.
2. Bluetooth is used for connecting to PC or mobile phone. To display the signals and measurements specially designed GUI is used in PC and for mobile phone (and also tablet) an android app is used. Motorola cell phone is providing an app developed with Java 2 Micro edition (J2ME).
3. IEEE 80215.4/ZigBee is used for PC connection. The signals can be displayed by using LabView or MATLAB GUI.
4. WiFi is used for connecting to PC or mobile phone. With a specially designed GUI developed in Java, the signals can be displayed on PC and mobile phone. Open source software (the app is written in X-code using object C) developed by E.P. Ltd. is available in Apple's iPhone 4S smartphone.
5. GSM/GPRS/GPS is used for long distance monitoring of ECG by connecting with PC or mobile phone.
6. Some of the system on chip (SoC) products like AT86RF212B, AT86RF233, AT86RF215, AT86RF215M, and AT86RF215IQ [142] will provide wireless communication network through ZigBee technology. TMS37157, TRF796X, TRF7970, AT86RF212, MCRF200, ADF7021, and ADF7025 [143] will provide communication using RFID technology. CC1101, CC1110, CC430, CC1190, CC11XL, CC112X, and CC120X [143] will provide communication using WPAN technology. CC2520, CC2530, CC2530ZNP, CC2531, CC2533, ADF4242, and AT86RF231 [143] will provide communication using ZigBee technology. CC2560, CC2540, CC2570, EM250, EM260, BCM4329, and BCM2045 [143] will provide communication using Bluetooth technology. WL1271, WL1281, BCM43241, BCM25/29, BCM4318, BCM4330, BCM4752, and AR6102 [143] will provide communication using WiFi technology. And WL1281, NL5500, UBX-G6010, BCM4750, and SiR starV [143] will provide communication using GPS technology.

#### 2.8.3. Selection of Effective Communication Technique

The communication mode is selected based on the distance of monitoring the signals. For short distance wired connection like USB SPI or wireless communication (1 or up to 100 m, depending on radio class) through Bluetooth or ZigBee technology (up to 75 m) or WiFi (indoors about 150 feet (46 m) and outdoors about 300 feet (92 m)) is generally preferred. For long distances GSM/GPRS (35 kilometres) or GPS (up to 25000 Km) is preferable. At present, all the communication technologies are inbuilt in the hardware and available as SoC (some of available SoC products are given in Section 2.8.2). When the SoC is selected for signal processing, it is better to select the suitable SoC product which is having preferred communication network technology. By providing long distance communication with the doctor, there is a scope for online monitoring of the patient condition and online diagnosis. This will not only save money and time, it will save lives of poor people. And also it helps to develop a smart city in the area of medical engineering.

## 3. Discussion

The death rate is increasing every year due to heart diseases from past few decades in India. This can be reduced by early detection of symptoms of abnormalities. A few years back, the ECG systems for detecting abnormalities were only available in the hospital and used only in the presence of specialists. It was very difficult to go every time to the hospital and take the ECG, which was also very expensive, especially for rural people. But present situation is slowly changing by using the health monitoring systems. Therefore, everything is going to change within few years in India like developed countries in the field of biomedicine by developing the smart and wearable health monitoring systems. So much of work is done by many people, but there is a lack of validation and communication provision with the doctor. There are options for recording and sending the data to the service centre where the data is analysed. But there is no accessibility of data to the user or patient. And also, they used commercial software which is licensed and very costly. Therefore, the net cost is very high.

In order to overcome these limitations and to add the missing features in existing systems, a new framework is proposed in this paper. In this review, quantitative information for designing of external cardiac loop recorder (ELR) is presented as a study of real-time ECG monitoring from remote area continuously. With the proper selection of the devices such as electrodes/sensors, instrumentation amplifier, filters, processor, and communication mode, an advanced external cardiac loop recorder is going to be designed to achieve better performance with less cost. New framework includes an option for saving the recorded ASCII data in text or excel form, and then it is easy to access and process the data. Further, the data can be processed and extract the features for detecting the normal or abnormal condition of the patient by using open source software called Scilab that reduces the cost of the system by avoiding commercial software used for analysis. And also by using open source software like TeraTerm, CoolTerm, and Processing with Arduino, data can be sent to a doctor via Bluetooth or Wi-Fi. Using Gobetwino open source software with Arduino data can be sent via the internet. Therefore, the doctor can receive and analyse the data using open source software and further he can send the suggestions or precautions to the patient at an early stage. If such a system is designed, it would become a milestone in the field of biomedical engineering and would help to develop the smart city towards the biomedical field in India. And also it will reach the rural people effectively so that the death rate due to heart diseases can be reduced.

It is evident from Tables 1 [Table tab2] [Table tab3] [Table tab4] [Table tab5] [Table tab6] [Table tab7] [Table tab8]–9 that one can design and configure appropriate internal circuitry components for the development of the cost effective external cardiac loop recorder system. The appropriate selection of open source software along with suitable internal circuitry will give way for new ELR suitable for implementation with less cost. Under a pilot process, a working prototype is under investigation by duly considering all the design parameters and software requirements. This expected design system will ensure the required diagnostic precision suitable for detecting the cardiac episodes.

## 4. Conclusion

This research study provided an insight into the systematic review on external cardiac loop recorders. It gives the quantitative information which helps in the selection of internal parts of the external cardiac loop recorder. Although several techniques for monitoring cardiac episodes were available, the scope for a new cardiac device is still in demand. This is due to the fact that the real-time cardiac episodes monitoring and its corresponding alert mechanism can help in saving the life of the patient. Such mechanism through the advent of cost effecting wearable external cardiac loop recorder will provide a major healthcare revolution in the developing countries.

## Figures and Tables

**Table 1:** Comparison among Holter monitor, ELR, and ILR.

	Advantages	Limitations	Indications	Diagnostic yield
Holter monitor	Low cost, continuous monitoring	Short duration of monitoring with low diagnostic yield	Patients with very frequent symptoms (≥1 week)	6–22%
External loop recorder	Retrospective and prospective ECG records, possibility to record asymptomatic arrhythmias automatically	Poor recordings, poor patient compliance to wearing device, continuous device maintenance required	Compliant patients with intersymptom interval ≤ 4 weeks	24–47%
Implantable loop recorder	Prolonged monitoring without external electrodes, highest diagnostic yield	Invasive implantation with risk of local complications, high cost	Early phase of evaluation of patients with recurrent syncope of uncertain origin that have absence of high risk criteria that require immediate hospitalization or intensive evaluation and a likely recurrence within device battery longevity	43–78%

**Table 2 tab2:** ILR & ELR products available.

Device/company	Mode	Expected monitoring duration	Max continuous recording period
Reveal Plus 9526/Medtronic	Implantable	14 months	—
Reveal DX/Medtronic	Implantable	3 years	42 min
Reveal XT/Medtronic	Implantable	3 years	42 min
Reveal LINQ/Medtronic	Implantable	3 years	—
Sleuth/Transoma	Implantable	28 months	630 min
Confirm DM2100/St. Jude	Implantable	3 years	48 min (147 episodes)
Confirm DM 2102/St. Jude	Implantable	3 years	48 min (147 episodes)
MCOT/CardioNet	External	Few weeks	21-day continuous monitoring
LifeStar ACT/LifeWatch	External	Few weeks	21-day retrievable monitoring
LifeStar/LifeWatch	External	Few weeks	10 min
eVolution/eCardio	External	Few weeks	30 min
3300 BT/Vitaphone	External	Few weeks	20 min
V-PATCH/Medical System	External	Few weeks	30 h
King of the Heart/Instrumedics	External	Few weeks	6 min
SpiderFlash/Sorin	External	Few weeks	Several hours
Cardiocall/Reynolds Esaote	External	Few weeks	18 min
Super/I-Cardia	External	Depends on patient compliance	2 recordings
Cardio PAL/Medicomp	External	Depends on patient compliance	—
SEEQ MCT/Medtronics	External	30 days	—
Piix NUVANT MCT/Corventis	External	7 days	—
HCG801/Omron	External	30 seconds can be made when symptoms occur	30 sec window indication, 125 MB memory required
SEER 1000/GE Healthcare	External	24 h or 48 h or 3 days (three modes are available)	Nonremovable digital memory
SEER Light/GE Healthcare	External	24 h (48 h for SEER Light extent)	32 MB memory required

**Table 3 tab3:** Specifications of some accelerometer ICs available.

Accelerometer IC	Supply voltage	Power consumption	Full scale range	Bandwidth
ADXL335	1.8 V–3.6 V	350 *μ*A (typical)	±3 g	For the *X*- and *Y*-axes 0.5 Hz to 1600 Hz and for the *Z*-axis 0.5 Hz to 550 Hz
ADXL330	2.0 V–3.6 V	200 *μ*A and VS = 2.0 V (typical)	±3 g	For *X*- and *Y*-axes 0.5 Hz to 1,600 Hz and for the *Z*-axis 0.5 Hz to 550 Hz
ADXL345	2.0 V–3.6 V	40 *μ*A at VS = 2.5 V (typical)	±16 g	
SDI1221	+5.0 and +2.5 volts	+5 VDC, 8 mA power (typical)	±2 g	0–400 Hz
SCA3000	2.35 V–3.6 V	2.5 V, 480 *μ*A typ	±2 g	45 Hz (typical)
LIS344ALH	2.4 V–3.6 V		±2 g/±6 g	1.8 kHz for all axes
MMA7260Q/ MMA7260QT	2.2 V–3.6 V	500 *μ*A	±1.5 g/2 g/4 g/6 g	350 Hz for *X* & *Y* and 150 Hz for *Z*
MMA8451Q	1.95 V–3.6 V	6 *μ*A to 165 *μ*A	±2 g/±4 g/±8 g	
Bosch BMA180	VDD = 1.62 V–3.6 V and VDDIO = 1.2 V–3.6 V	650 *μ*A (typical)	±1 g, ±1.5 g, ±2 g, ±3 g, ±4 g, ±8 g, ±16 g	0.2 Hz–300 Hz for BPF

**Table 4 tab4:** Specifications of some gyro ICs.

Ref. number	Gyro IC/sensor	Operating voltage	Axes
[17]	L3G4200D	2.6 V–5.5 V	±250 (*X*), ±500 (*Y*), ±2000°/s (*Z*)
[18]	ENC03J	2.7 V–5.5 V	Max ±300°/s
[19]	ENV05S	8–13.5 V	Max ±90°/s
[20]	Integrated Dual-Axis Gyro-IDG-300	3 V–3.5 V	Full scale range of ±500°/sec
[21]	Integrated Dual-Axis Gyro-IDG-500	2.7 V–3.3 V	Full scale range of ±500°/sec
[22]	Single Chip Rate Gyro EVAL-ADXRS610	4.75 V–5.25 V (typical 5 V)	±300°/sec yaw rate
[23]	SCC2000 Series Combined Gyro Sensor and Accelerometer	3 V–3.6 V	*X*- or *Z*-axis ±125°/s or ±300°/s
[24]	XV-3500CB/XV3900CB	3.3 V	±100°/s
[24]	XV-3510CB	3.3 V	±300°/s
[24]	XV-3700CB	3.3 V	±300°/s to ±1500°/s
[24]	XV7011BB/XV7001BB	2.7 V to 3.6 V	±100°/s
[24]	AH-6120LR	3 V	±1000°/s
[24]	AP-6110LR	2.85 V to 3.6 V	±300°/s

**Table 5 tab5:** Differences between gyroscope and accelerometer.

S. number	Gyroscope	Accelerometer
1	It determines orientation	It measures static (e.g., gravity) as well as dynamic (e.g., sudden starts/stops) acceleration
2	Senses rotation	Cannot sense rotation
3	It measures the rotation rate around a particular axis based on angular momentum	It measures linear acceleration based on vibration
4	A gyroscope is used to determine angular position	Two-axis accelerometer is used to determine the direction of gravity
5	Applications: in navigation on unmanned aerial vehicles, compasses and large boats, ultimately assisting with stability in navigation, and altitude; indicator on typical aircraft	Applications: determines screen orientation and acts as a compass undoing actions by simply shaking the smartphone
6	Gyroscopes are used in extra earth navigation (spacecraft), where the planet earth's pull and influence disappear	3-axis accelerometer could identify the orientation of an object relative to the Earth's surface

**Table 6 tab6:** Electrode placement for different type of wearable.

Ref. paper	Wearable type	Number of electrodes	Type of electrodes	Placement of electrodes
[25]	Tight fitted sleeveless top	—	Dry Ag/AgCl electrode	Chest line

[26]	Wearable (vital jacket system)	—	—	On chest

[27]	BioShirt	3	3 M Ag/AgCl 2223 monitoring electrode which has foam tape and sticky gel	ECG limb leads and augmented unipolar limb leads

[28]	Belt type	2	ECG	RA-LA 11 cm apart through midline on chest

[29]	Wearable belt	4	ECG	Channel 1 (+), in the fifth intercostal space in anterior axillary line. Channel 1 (−), manubrium of sternum on the right side. Channel 2 (+), on sternum on the same altitude as the fourth intercostal space. Channel 2 (−), left subclavian area. Ground: in the fifth intercostal space in midaxillary line

[30]	Wearable chest harness	—	Coin sized dry-contact electrodes	On chest

[31]	Wearable chest belt	2	ECG	On chest

[32]	Chest belt	2		On chest

[33]	Wearable ECG vest	3	Ag-AgCl	Three Velcro tapes in neck, back, and waist

[34]	Wearable	3	Ag/AgCl	LA, RA, RF

[35]	Wearable	3		RA-LA 5 cm through midline and LL-LA end to center of LL 6 cm down

[36]	Wearable	3		Einthoven triangle

[37]		3	ECG	RA-RL-LA placed b/w midline & distance RA-LA is 5 cm. LL is 5 cm down from RA-LA line and 5 cm left from midline

[38]	—	—	—	Sensors on the lumbar support cushion of the seat

[39]	—	10	—	Standard positions to generate 12 leads

[40]	—	3	—	Einthoven triangle

[41]	—	3	ECG	RA-LA-RL

[42]	—	—	Dry clamp electrodes	Located on the wrists

[43]	—	2	Capacitive coupling electrodes	On chest lead I

[44]	—	12	—	12-lead ECG system

[45]	—	—	QUASAR's capacitive bioelectrodes (can measure with clothes)	Integrated into a pad system that is placed over a chair

[46]	—	12	—	12-lead standard placement

[47]	—	3	—	Einthoven triangle

[48]	—	—	Patch-type electrode	On chest

[49]	—	12	—	12-lead standard placement

[50]	—	—	Wet gel Ag/AgCl electrodes (Ambu, Blue Sensor R)	Below the left pectoral muscle

[51]	—	3	—	(RA, LA, RL), lead II

[52]	Not wearable	3	—	LA, RA, LF (separated by 10 cm) and an extra electrode placed on RL (forms an equilateral triangle)

[53]	Not wearable	4	—	RA, LA, LL, RL

**Table 7 tab7:** CMRR comparison of different works done for ECG amplifier using CMOS technology.

Reference paper	CMRR	Process tech	Battery voltage
[54]	71 dB	0.18 *μ*m	1.8 V dual
[55]	141.61 dB	0.18 *μ*m	1.8 V dual
[56]	82 dB	0.18 *μ*m	—
[57]	>125 dB	0.18 *μ*m	0.4 V
[58]	62 dB	—	3.3 V
[59]	>100 dB	—	3.3 V
[60]	80 dB	0.13 *μ*m	0.7 V
[61]	150 dB	0.18 *μ*m	1.8 V
[62]	167.87 dB	—	—
[63]	125 dB	0.18 *μ*m	—

**Table 8 tab8:** Filters and their frequency range for various ECG parameters.

Ref. number	Parameters acquired	Filter used	Freq range
[64]	Heart rate	Bandpass filter	—
[30]	Heart rate	Passive RC high pass filter	1 Hz
[31]	Heart rate	LPF, after IA notch, HPF, LPF	*F*_lpf_ = 150 Hz, *F*_*n*_ = 60 Hz, *F*_hpf_ = 0.5 Hz, *F*_lpf_ = 35 Hz
[65]	Heart rate	LPF	*F*_lpf_ = 40–80 Hz
[45]	Heart rate	8-pole Bessel bandpass filter	0.1–100 Hz
[37]	QRS complexes, heart rate	BPF	
[28]	R-peak, heart rate	HPF, 2nd-order Butterworth filter (two 1st-order LPF)	*F*_*h*_ = 0.05 Hz, *F*_*L*_ = 35 Hz
[32]	R-peak, abnormal heart beat	LPF, moving average filter	*F*_lpf_ = 35 Hz
[66]	ECG and heart rate	Notch filter formed by ordinary amplifier TL062	*F*_*c*_ = 50 Hz
[38]	ECG wave, R-peak	LPF, BPF	*F*_bpf_ = 5–20 Hz
[67]	R-peaks	LPF, HPF	
[44]	R-peak	Adaptive filter	
[68]	Pk-Pk	Analog active RC filter, a second-order Butterworth	
[29]	HRV	LPF	
[42]	ECG, PPG, BP	HPF, LPF	*F*_*h*_ = 0.16 Hz, *F*_*l*_ = 103 Hz
[69]	QRS complex	Antialiasing 1-pole LPF	*F*_*l*_ = 35 Hz,
[70]	QRS, T wave	HPF, sixth-order Bessel LPF	*F*_lpf_ = 150 Hz
[50]	QRS complexes and T waves	RC high pass filters	*F*_3-dB_ = 0.16 Hz
[71]	PQRST wave	Bandpass filter	0.159–159 Hz
[72]	Points (P, Q, R, S, T)	BPF, notch filter	*F*_bpf_ = 0.05 Hz to 150 Hz, *F*_*n*_ = 6 Hz
[51]	QRS duration, RR interval, HBR, R amplitude, RT-interval: PR-interval: QT-interval features	LPF, HPF, LPF	*F*_lpf_ = 0.03 Hz, *F*_hpf_ = 80 Hz, *F*_lpf_ = 58 Hz and 19 Hz

**Table 9:** Different microcontrollers used for ECG monitoring.

MP or MC used	Supply voltage range	Max power consumption	Memory storage
MSP430	2.5 V to 5.5 V	330 *μ*A at 1 MHz, 3 V	2 k byte ROM, 128-byte RAM
MSP430F5529	1.8 V to 3.6 V	290 *μ*A at 8 MHz, 3.0 V	128 KB flash & 8 × 2 KB SRAM
MSP430F5419A	1.8 V to 3.6 V	230 *μ*A at 8 MHz, 3.0 V	128 KB flash & 16 KB SRAM
MSP430F5515	1.8 V to 3.6 V	290 *μ*A at 8 MHz, 3.0 V	64 KB flash & 4 × 2 KB SRAM
MSP430 (F2)	1.8 V to 3.6 V	220 *μ*A at 1 MHz, 2.2 V	1 KB + 256 B flash memory 128 B RAM
MSP430F1232	1.8 V–3.6 V	200 *μ*A at 1 MHz, 2.2 V	8 KB + 256 B flash memory, 256 B RAM
MSP430FG439	1.8 V to 3.6 V	300 *μ*A at 1 MHz, 2.2 V	60 KB + 256 B flash memory, 2 KB RAM
MSP430F2418	1.8 V to 3.6 V	365 *μ*A at 1 MHz, 2.2 V	116 KB + 256 B flash memory, 8 KB RAM
TI CC2530	2 V–3.6 V	29 mA at 2.4 GHz	32 KB flash & 8 KB RAM
TI CC2431	2 V–3.6 V	27 mA at 32 MHz	128 KB flash & 8 KB RAM
TMS320VC5509A	2.7-V–3.6-V	—	128 K × 16-bit on-chip RAM, 64 K bytes one wait state on-chip ROM, 16 MB DRAM
TMS320F2812	1.8 V–3.3 V	1.9-V Core at 150 MHz	128 K × 16 flash, 128 K × 16 ROM
TMDX5505eZDsp/VC5505eZdsp	1.8 V, 2.5 V, 2.8 V, 3.3 V	—	320 KB of on-chip RAM, 128 KB of on-chip ROM
ATmega8	4.5 V–5.5 V	3.6 mA at 4 MHz, 3 V, 25°C	8 KB flash, 512 B EEPROM, 1 KB SRAM
ATmega8L	2.7 V–5.5 V	3.6 mA at 4 MHz, 3 V, 25°C	8 KB flash, 512 B EEPROM, 1 KB SRAM
ATmega328	1.8–5.5 V	0.2 mA at 1 MHz, 1.8 V, 25°C	32 KB of flash, 1 K byte EEPROM, 2 KB of SRAM
Arduino (ATmega328)	5 V	—	32 KB of flash, 1 K byte EEPROM, 2 KB of SRAM
Concerto MCU (MB95F108AHS)	5 V	—	60 KB dual-flash, 2 KB RAM
PIC18LF4620	2.0 V to 5.5 V	—	64 KB flash, 3968 SRAM, 1024 EEROM
ADuC842	—	4.5 mA at 3 V (core CLK = 2.098 MHz)	64 KB flash, 2 KB SRAM
C8051F021	2.7 V–3.6 V	—	4.25 KB RAM, 64 KB ROM
32-bit ARM cortex M0 CPU	—	64.3 *μ*W/MHz	—

## References

[1] Zimetbaum P., Goldman A. (2010). Ambulatory arrhythmia monitoring. *American Heart Association, Circulation*.

[2] http://www.heart.org/HEARTORG/Conditions/HeartAttack/SymptomsDiagnosisofHeartAttack/Holter-Monitor_UCM_446437_Article.jsp#.V_o8kk-LXnM

[3] http://stuccu.com/s/Holter+Monitor-MbSLsTI-Buy-Exclusive-Deals-70-OFF-Save-Big- Lowest-Price-on-Holter-Monitor

[4] http://www.medhelp.org/posts/Heart-Rhythm/Cost-for-holtor-monitor/show/1630319

[5] http://wwwp.medtronic.com/Newsroom/LinkedItemDetails.do?itemId=1160041295600%20&%20format=pdf%20&lang=en_IN

[6] Krahn A. D., Klein G. J., Yee R., Hoch J. S., Skanes A. C. (2003). Cost implications of testing strategy in patients with syncope: randomized assessment of syncope trial. *Journal of the American College of Cardiology*.

[7] http://www.ispor.org/ScientificPresentationsDatabase/Presentation/54447

[8] Subbiah R., Chia P.-L., Gula L. J. (2013). Cardiac monitoring in patients with syncope: making that elusive diagnosis. *Current Cardiology Reviews*.

[9] Nachane C., Subramanian D., Warrier J., Sinha V. (2015). Development of acquisition of ECG during treadmill exercise. *International Journal of Scientific & Engineering Research*.

[10] Brignole M., Vardas P., Hoffman E. (2009). Indications for the use of diagnostic implantable and external ECG loop recorders. *Europace*.

[11] http://www3.gehealthcare.co.uk

[12] http://www3.gehealthcare.pl/~/media/downloads/uk/product/diagnostic%20ecg/ambulatory/seer1000/ dcar_emea_brochure_seer_1000_with_cardioday_english_doc1286154_rev2_11-2013.pdf?Parent=%7BF194EDD5-D167-469C-B6A3-E21B8ABE8393%7D

[13] http://www.medtronicdiagnostics.com

[14] http://www.vicare-medical.dk/admin/UploadFile.aspx?path=/UserUploadFiles/ Monitorering/ Corventis %20Event%20 recorder/ Nuvant_Spec.pdf

[15] http://omronhealthcare.com.au/pdf2/HCG-801_Brochure.pdf

[16] http://www.mrisafety.com/SafetyInfov.asp?SafetyInfoID=249

[17] Tanaka S., Motoi K., Nogawa M., Yamakoshi K. A new portable device for ambulatory monitoring of human posture and walking velocity using miniature accelerometers and gyroscope.

[18] Tanaka S., Motoi K., Nogawa M., Yamakoshi K. A new portable device for ambulatory monitoring of human posture and walking velocity using miniature accelerometers and gyroscope.

[19] Singh A. K., Gorain U. K. (2004). Development of vibrating disc piezoelectric gyroscope. *Defence Science Journal*.

[20] https://www.sparkfun.com/datasheets/Components/IDG-300_Datasheet.pdf

[21] https://www.sparkfun.com/datasheets/Components/SMD/Datasheet_IDG500.pdf

[22] http://www.analog.com/media/en/technical-documentation/data-sheets/ADXRS610.pdf

[23] http://www.murata.com/en-eu/products/sensor/gyro/scc2000

[24] http://www5.epsondevice.com/en/products/standard_gyro/

[25] Cho H., Lee J. H. (2015). A study on the optimal positions of ECG electrodes in a garment for the design of ECG-monitoring clothing for male. *Journal of Medical Systems*.

[26] Zhang K., Song L., Lu D. Design of remote ECG monitoring system based on GPRS.

[27] Jang Y., Noh H. W., Lee I. B., Song Y. A basic study for patch type ambulatory 3-electrode ECG monitoring system for the analysis of acceleration signal and the limb leads and augmented unipolar limb leads signal.

[28] Kim B.-H., Noh Y.-H., Jeong D.-U. A wearable ECG monitoring system using adaptive EMD filter based on activity status.

[29] Altun A. A., Başçıfcı N. A wireless sensor network based on zigbee for ECG monitoring system.

[30] Valchinov E., Antoniou A., Rotas K., Pallikarakis N. Wearable ECG system for health and sports monitoring.

[31] Yap J. H., Jeong D. U. (2013). Design and implementation of ubiquitous ECG monitoring system by using android tablet. *Ubiquitous Information Technologies and Applications*.

[32] Noh Y.-H., Huei Y. J., Jeong D.-U. Implementation of the abnormal ECG monitoring system using heartbeat check map thechnique.

[33] Weiya W., Li G., Zhanfeng L., Gui H. Research on wearable EeG monitoring system based on ZigBee.

[34] Rosu M.-C. Implementation for a WBAN-ECG monitoring system (Preliminary results).

[35] Wang Y., Wunderlich R., Heinen S. Design and evaluation of a novel wireless reconstructed 3-lead ECG monitoring system.

[36] Acharyya A., Maharatna K., Al-Hashimi B. M., Tudugalle H. Simplified logic design methodology for fuzzy membership function based robust detection of maternal modulus maxima location: a low complexity Fetal ECG extraction architecture for mobile health monitoring systems.

[37] Wang Y., Doleschel S., Wunderlich R., Heinen S. (2015). A wearable wireless ECG monitoring system with dynamic transmission power control for long-term homecare. *Journal of Medical Systems*.

[38] Son J., Kim B., Park M. Lumbar cushion based real-time ECG sensing system for monitoring driver's state.

[39] Gaxiola-Sosa J. E., Mohsin N., Palliyali A. J., Tafreshi R., Entesari K. A portable 12-lead ECG wireless medical system for continuous cardiac-activity monitoring.

[40] Hadjem M., Salem O., Naït-Abdesselam F. An ECG monitoring system for prediction of cardiac anomalies using WBAN.

[41] Harmah D. J., Kathirvelu D. An ubiquitous miniaturized android based ECG monitoring system.

[42] Martinho J., Prates L., Costa J. (2014). Design and implementation of a wireless multiparameter patient monitoring system. *Procedia Technology*.

[43] Ping Z., Zhoucheng L., Feng W., Hongyu J. (2013). Non-contact ECG monitoring based on capacitive electrodes. springer, world congress on medical physics and biomedical engineering. *World Congress on Medical Physics and Biomedical Engineering May 26–31, 2012, Beijing, China*.

[44] Tse Z., Dumoulin C., Clifford G. (2012). Cardiac MRI with concurrent physiological monitoring using MRI-compatible 12-lead ECG. *Journal of Cardiovascular Magnetic Resonance*.

[45] McDonald N. J., Anumula H. A., Duff E., Soussou W. Noncontact ECG system for unobtrusive long-term monitoring.

[46] Chen T., Mazomenos E., Maharatna K., Dasmahapatra S., Niranjan M. On the trade-off of accuracy and computational complexity for classifying normal and abnormal ECG in remote CVD monitoring systems.

[47] Juan Pablo Tello P., Manjarres O., Quijano M., Ulises Blanco A. Remote monitoring system of ECG and temperature signals using Bluetooth.

[48] Fernández-López H., Correia J. H., Simões R., Afonso J. A. (2011). Experimental evaluation of IEEE 802.15.4/ZigBee for multi-patient ECG monitoring. *Electronic Healthcare*.

[49] Smoleń M., Kańtoch P., Augustyniak P., Kowalski P. (2012). Wearable patient home monitoring based on ECG and ACC sensors. *5th European Conference of the International Federation for Medical and Biological Engineering*.

[50] Lekkala J., Salpavaara T., Verho J., Riistama J. (2010). Simple inductively coupled resonance sensor for ECG and heart rate monitoring. *Procedia Engineering*.

[51] Gupta G. PC based ECG monitoring system.

[52] Kim M. S., Cho Y. C., Seo S.-T., Son C.-S., Kim Y.-N. (2011). Auto-detection of R wave in ECG (electrocardiography) for patch-type ECG remote monitoring system. *Biomedical Engineering Letters*.

[53] Loewe A., Schulze W. H. W., Jiang Y., Wilhelms M., Dössel O. (2011). Determination of optimal electrode positions of a wearable ECG monitoring system for detection of myocardial ischemia: a simulation study. *Computing in Cardiology*.

[54] Lau J. G., Marzuki A. B. (2014). A low power low noise CMOS amplifier for portable ECG monitoring application. *ARPN Journal of Engineering and Applied Sciences*.

[55] Dangi J., Gurjar R. C. An Ecg instrumentation amplifier with improved Cmrr and gain using. 18 *µ*m technology.

[56] Moni D. J., Gopalakrishnan N. (2013). A low power CMOS electrocardiogram amplifier design using 0.18 *μ*m CMOS technology. *International Journal of Advancements in Research & Technology*.

[57] Tseng Y., Ho Y., Kao S., Su C. (2012). A 0.09 W low power front-end biopotential amplifier for biosignal recording. *IEEE Transactions on Biomedical Circuits and Systems*.

[58] Ren M. Y., Zhang C. X., Sun D. S. Design of CMOS instrumentation amplifier.

[59] Xiu L., Li Z. Low-power instrumentation amplifier IC design for ECG system applications.

[60] Um J.-Y., Sim J.-Y., Park H.-J. (2010). A gate-leakage insensitive 0.7-V 233-nW ECG amplifier using non-feedback PMOS pseudo-resistors in 0.13-*μ*m N-well CMOS. *Journal of Semiconductor Technology and Science*.

[61] Wang W.-S., Wu Z.-C., Huang H.-Y., Luo C.-H. Low-power instrumental amplifier for portable ECG.

[62] Almazan S. P., Alunan L. I., Gomez F. R., Jarillas J. M., Gusad M. T., Rosales M. Monolithic CMOS current-mode instrumentation amplifiers for ECG signals.

[63] Nanda C., Mukhopadhyay J., Mandai D., Chakrabarti S. A CMOS instrumentation amplifier with low voltage and low noise for portable ECG monitoring systems.

[64] Liou J.-C., Shih T.-T., Lin W.-C., Huang Y.-C. Noninvasive ECG and EMG Electrode system for Health Monitoring and Science technology application.

[65] Shebi Ahammed S., Pillai B. C. (2013). Design of Wi-Fi based mobile Electrocardiogram monitoring system on concerto platform. *Procedia Engineering*.

[66] Chiu R.-D., Wu S.-H. A BAN system for realtime ECG monitoring : from wired to wireless measurements.

[67] Gao H., Duan X., Guo X., Huang A., Jiao B. Design and tests of a smartphones-based multi-lead ECG monitoring system.

[68] Rosu M.-C. Preliminary evaluation for an ECG monitoring system.

[69] Zeng M., Chung I.-Y., Lee J.-A., Lee J.-G. An on-node intelligence based energy efficient ECG monitoring system.

[70] Komensky T., Jurcisin M., Ruman K., Kovac O., Laqua D., Husar P. Ultra-wearable capacitive coupled and common electrode-free ECG monitoring system.

[71] Özkaraca O., Işik A. H., Güler I. Detection, real time processing and monitoring of ECG signal with a wearable system.

[72] Islam M. K., Shoeb M. A., Ahammad T., Nowrin T. F. Embedded programmable web-based ECG monitoring & detection system using a fast algorithm.

[73] http://www.skintact.com/

[74] http://www.medgadget.com

[75] http://www.meddeviceonline.com

[76] Baek J.-Y., An J.-H., Choi J.-M., Park K.-S., Lee S.-H. (2008). Flexible polymeric dry electrodes for the long-term monitoring of ECG. *Sensors and Actuators, A: Physical*.

[77] Gruetzmann A., Hansen S., Müller J. (2007). Novel dry electrodes for ECG monitoring. *Physiological Measurement*.

[78] Matthews R., McDonald N. J., Fridman I., Hervieux P., Nielsen T. Nonintrusive, wearable bioelectrodes for monitoring the heart and brain. http://www.sensorsmag.com/specialty-markets/medical-devices/nonintrusive-wearable-bioelectrodes-monitoring-heart-and-bra-1412.

[79] Sharma H. W., Singh M. (2014). Design and development of heart rate monitoring device with reduction of motion artefact using 3-axis accelerometer. *International Journal of Emerging Technology and Advanced Engineering*.

[80] Lin C.-H., Chen S.-Y., Yang C.-C. Structural health monitoring of bridges using cost-effective 1-axis accelerometers.

[81] Gjoreski H., Rashkovska A., Kozina S., Luštrek M., Gams M. Telehealth using ECG sensor and accelerometer.

[82] Dhivya Poorani V., Ganapathy K., Vaidehi V. Sensor based decision making inference system for remote health monitoring.

[83] Kantoch E., Smolen M., Augustyniak P., Kowalski P. (2011). Wireless body area network system based on ECG and accelerometer pattern. *Computing in Cardiology*.

[84] Dinh A. Heart activity monitoring on smartphone.

[85] Lee J., Jung J., Lee J., Kim Y. T. Diagnostic device for acute cardiac disease using ECG and accelerometer.

[86] Penders J., Altini M., van de Molengraft J., Yazicioglu F., Van Hoof C. A low-power wireless ECG necklace for reliable cardiac activity monitoring on-the-move.

[87] Augustyniak P., Smoleń M., Mikrut Z., Kańtoch E. (2014). Seamless tracing of human behavior using complementary wearable and house-embedded sensors. *Sensors*.

[88] Khan A. M., Siddiqi M. H., Lee S.-W. (2013). Exploratory data analysis of acceleration signals to select light-weight and accurate features for real-time activity recognition on smartphones. *Sensors*.

[89] Muaremi A., Seiter J., Tröster G., Bexheti A. Monitor and understand pilgrims: data collection using smartphones and wearable devices.

[90] Shi W. V., Zhou M. Recent advances of sensors for pacemakers.

[91] Grosse-Puppendahl T., Berlin E., Borazio M. (2012). Enhancing accelerometer-based activity recognition with capacitive proximity sensing. *Ambient Intelligence*.

[92] Poh M.-Z., Swenson N. C., Picard R. W. (2010). Motion-tolerant magnetic earring sensor and wireless earpiece for wearable photoplethysmography. *IEEE Transactions on Information Technology in Biomedicine*.

[93] Preece S. J., Goulermas J. Y., Kenney L. P. J., Howard D. (2009). A comparison of feature extraction methods for the classification of dynamic activities from accelerometer data. *IEEE Transactions on Biomedical Engineering*.

[94] Jafari Tadi M., Koivisto T., Pänkäälä M., Paasio A. (2014). Accelerometer-based method for extracting respiratory and cardiac gating information for dual gating during nuclear medicine imaging. *International Journal of Biomedical Imaging*.

[95] Chan A. M., Ferdosi N., Narasimhan R. Ambulatory respiratory rate detection using ECG and a triaxial accelerometer.

[96] Liu G.-Z., Guo Y.-W., Zhu Q.-S., Huang B.-Y., Wang L. (2011). Estimation of respiration rate from three-dimensional acceleration data based on body sensor network. *Telemedicine Journal and e-Health*.

[97] Okada Y., Yoto T. Y., Suzuki T., Sakuragawa S., Sugiura T. Wearable ECG recorder with acceleration sensors for monitoring daily stress: office work simulation study.

[98] Oudre L., Lung-Yut-Fong A., Bianchi P. Segmentation of accelerometer signals recorded during continuous treadmill walking.

[99] Sejdić E., Lowry K. A., Bellanca J., Redfern M. S., Brach J. S. (2014). A Comprehensive Assessment of Gait Accelerometry Signals in Time, Frequency and Time-Frequency Domains. *IEEE Transactions on Neural Systems and Rehabilitation Engineering*.

[100] Studnička F., Šeba P., Jezbera D., Kříž J. Continuous monitoring of heart rate using accelerometric sensors.

[101] Bhuyan A. I., Mallick T. C. Gyro-accelerometer based control of a robotic arm using AVR microcontroller.

[102] Chen Y., Oliveira J. M., Hunter I. W. Sensor architecture for a two-actuator robotic endoscope tip.

[103] Liu C., Wang Z. Design and realization of data acquiring system for vehicle speed sensor and gyroscope.

[104] Li G., Wang F., Xiao G., Wei G., Zhang P., Long X. (2015). Temperature compensation method using readout signals of ring laser gyroscope. *Optics Express*.

[105] Xia D., Chen S., Wang S. (2009). Development of a prototype miniature silicon microgyroscope. *Sensors*.

[106] Chen X., Hu X., Ren R. Noninvasive ambulatory monitoring of the electric and mechanical function of heart with a multifunction wearable sensor.

[107] Brzostowski K., Drapala J., Swiatek J. (2013). Data-driven models for eHealth applications. *International Journal of Computer Science and Artificial Intelligence*.

[108] Khazraee M., Zamani A. R., Hallajian M. A novel hardware implementation for joint heart rate, respiration rate, and gait analysis applied to body area networks.

[109] Gargiulo G., Bifulco P., Cesarelli M. (2010). An ultra-high input impedance ECG amplifier for long-term monitoring of athletes. *Medical Devices: Evidence and Research*.

[110] Sullivan T. J., Deiss S. R., Cauwenberghs G. A low-noise, non-contact EEG/ECG sensor.

[111] Gomez-Clapers J., Serrano-Finetti E., Casanella R., Pallas-Areny R. Can driven-right-leg circuits increase interference in ECG amplifiers?.

[112] Bhat A. Q., Kumar V., Kumar S. (2013). Design of ECG data acquisition system. *International Journal of Advanced Research in Computer Science and Software Engineering*.

[113] Wang K., Ma S., Feng J., Zhang W., Fan M., Zha D. Design of ECG signal acquisition system based on DSP.

[114] Richard E., Chan A. D. C. Design of a gel-less two-electrode ECG monitor.

[115] Krishnan J., Khambete N. D., Rajan A., Benjamin B. (2013). Low power multiparameter biopotential amplifier system. *International Journal of Science and Research*.

[116] Burke M., Jassambo C. (2010). An ultra-low power dry-electrode ECG amplifier having optimized low-frequency response and CMRR. *Recent Researches in Circuits and Systems*.

[117] Assambo C., Burke M. J. Amplifier input impedance in dry electrode ECG recording.

[118] Inan O. T., Kovacs G. T. A. (2010). An 11 *μ* w, two-electrode transimpedance biosignal amplifier with active current feedback stabilization. *IEEE Transactions on Biomedical Circuits and Systems*.

[119] Spinelli E. M., Pallàs-Areny R., Mayosky M. A. (2003). AC-coupled front-end for biopotential measurements. *IEEE Transactions on Biomedical Engineering*.

[120] Bai Y.-W., Cheng C.-Y., Lu C.-K., Huang C.-H., Chen Y.-T., Lin Y.-N. Adjustable 60 Hz noise reduction and ECG signal amplification of a remote electrocardiogram system.

[121] Song S., Rooijakkers M., Harpe P. (2015). A low-voltage chopper-stabilized amplifier for fetal ECG monitoring with a 1.41 power efficiency factor. *IEEE Transactions on Biomedical Circuits and Systems*.

[122] Tu C.-C., Lin T.-H. Analog front-end amplifier for ECG applications with feed-forward EOS cancellation.

[123] Wang S., Wang Y., Chen L. A 192nW inverter-based chopper instrumentation amplifier for micropower ECG applications.

[124] Zhang J., Wang L., Li B. (2009). Design of low-offset low-power CMOS amplifier for biosensor application. *Journal of Biomedical Science and Engineering*.

[125] Rowlands D., James D. A., Vanegas C., Rao S., Lisner P. Design and fabrication of an ECG amplifier on silicon using standard CMOS process.

[126] Lee B., Higman T. 1.2V constant-gm rail-to-rail CMOS Op-Amp input stage with new overlapped transition regions technique for ECG amplifier.

[127] Ghamati M., Maymandi-Nejad M. A low-noise low-power MOSFET only electrocardiogram amplifier.

[128] Wu C.-C., Kuo W.-C., Wang H.-J. A pliable and batteryless real-time ECG monitoring system-in-a-patch.

[129] Pani D., Dessì A., Saenz-Cogollo J. F., Barabino G., Fraboni B., Bonfiglio A. (2016). Fully textile, PEDOT:PSS based electrodes for wearable ECG monitoring systems. *IEEE Transactions on Biomedical Engineering*.

[130] http://www.ti.com

[131] http://www.analog.com

[132] Ribeiro D. M. D., Colunas M. F. M., Marques F. A. F., Fernandes J. M., Cunha J. P. S. A real time, wearable ECG and continous blood pressure monitoring system for first responders.

[133] Wang K., Ma S., Feng J., Zhang W., Fan M., Zhao D. (2012). Design of ECG signal acquisition system based on DSP. *Procedia Engineering*.

[134] Chi J. N., Yan Y. T., Liu M. C., Yang L. The development of a Portable ECG monitor based on DSP.

[135] Chai J. The design of mobile ECG monitoring system.

[136] Yang G., Cai X., Wang F., Cu S., Zhao L. (2011). Research of portable ECG monitoring device. *Advances in Computer, Communication, Control and Automation*.

[137] Campillo D., Torres H., Gonzalez R., Valdes K., Lopez R. (2014). A portable device for a modular system of patient ECG monitoring. *Computing in Cardiology*.

[138] Ken C., Xiaoying L. A Zigbee based mesh network for ECG monitoring system.

[139] Alzate E. B., Martinez F. M. ECG monitoring system based on ARM9 and mobile phone technologies.

[140] Shin W., Cha Y. D., Yoon G. (2010). ECG/PPG integer signal processing for a ubiquitous health monitoring system. *Journal of Medical Systems*.

[141] Guo X., Chen W., Xu X., Li H. The research of portable ECG monitoring system with USB host interface.

[142] http://www.atmel.com/products/smart-energy/wireless-communications/default.aspx

[143] https://www.lsr.com/white-papers/soc-vs-sdr-for-wireless-product-design

